# Prediabetes: A precursor of diabetes and harbinger of premature CVD

**DOI:** 10.1016/j.ajpc.2026.101518

**Published:** 2026-03-02

**Authors:** Ritika Dhruve, Ambarish Pandey, Neil Keshvani

**Affiliations:** a Division of Cardiology, Department of Medicine, University of Texas Southwestern, Dallas, TX, USA; b Baylor Scott and White Research Institute, Dallas, TX, USA; c Baylor Scott and White The Heart Hospital, Plano, TX, USA

**Keywords:** Prediabetes, Obesity, ASCVD

Over the past several decades, substantial investment in identifying and treating traditional cardiovascular risk factors has yielded meaningful reductions in atherosclerotic cardiovascular disease (ASCVD) morbidity and mortality. Widespread statin use, improved blood pressure control, and sustained public health campaigns targeting tobacco use have collectively contributed to declining age-adjusted rates of myocardial infarction and stroke in high-income countries. Despite these advances, ASCVD remains a leading cause of death globally, accounting for an estimated 17.9 million deaths annually, and the pace of further event reduction has stagnated, particularly among younger and middle-aged adults [1]. This plateau suggests that contemporary prevention frameworks, which were largely developed around hyperlipidemia, hypertension, and smoking, are incomplete. Increasingly, attention has turned toward the metabolic determinants of cardiovascular risk, specifically, the interrelated conditions of obesity, insulin resistance, and dysglycemia, as key contemporary drivers of ASCVD risk. To meaningfully reduce the burden of cardiovascular events in the coming decades, a greater emphasis must be placed on identifying and addressing these upstream metabolic risk factors.

Type 2 diabetes is well established as an ASCVD risk enhancer and is embedded in structured management pathways that include glycemic monitoring, pharmacotherapy, and cardiovascular risk reduction. However, insulin resistance does not begin at the diagnostic threshold for diabetes but instead exists on a continuum, and the cardiovascular consequences of dysglycemia may accumulate long before a hemoglobin A1c (HbA1c) of 6.5 % is reached. The growing burden of insulin resistance in the United States underscores this concern. Recent national estimates indicate that 16 % of U.S. adults now have diabetes; however, for every adult living with diabetes, roughly three more harbor prediabetes, defined as HbA1c of 5.7–6.4 % or a fasting plasma glucose of 100–125 mg/dL [2]. Furthermore, the overlap with obesity is extensive, with over 80 % of individuals with prediabetes having overweight or obesity [3].

Despite this burden, prediabetes occupies an uncertain position in cardiovascular risk management. Unlike type 2 diabetes, for which considerable attention and intervention have been directed toward cardiovascular risk reduction, prediabetes is frequently documented passively through routine laboratory results without prompting meaningful clinical action. This clinical inertia persists despite meta-analyses of over 10 million individuals demonstrating that prediabetes independently confers an increased risk of ASCVD compared to normoglycemia [4]. However, critical knowledge gaps remain. Prior studies establishing the prediabetes-ASCVD association were largely conducted in older or more selective cohorts, and the extent to which this risk varies across the adult lifespan or is modified by concurrent obesity in a contemporary, diverse population has not been well characterized.

The study by Rana et al., published in this issue of the *Journal*, addresses these knowledge gaps [5]. The investigators conducted a retrospective cohort analysis of 1358,882 adults within the Kaiser Permanente Northern California integrated healthcare system who received care from 2015 to 2018 and who were free of prevalent diabetes or ASCVD at baseline. Prediabetes was ascertained through a combination of ICD-9/10-CM codes and laboratory values. The primary outcome was a composite of incident myocardial infarction, stroke, or revascularization over a mean follow-up of 4.1 years. After adjustment, prediabetes was independently associated with a 21 % increased risk of incident ASCVD (hazard ratio [HR] 1.21, 95 % CI, 1.18–1.25). The effect estimates are broadly concordant with the pooled relative risk of 1.15 for composite cardiovascular disease in prior studies [4]. Notably, these associations were not uniform across the lifespan. The relative risk was highest among young adults aged 18–34 (HR 1.54, 95 % CI, 1.18–2.02) and attenuated progressively with increasing age. However, while the relative risk was most pronounced in younger adults, the absolute event rate in this group was low (0.37 per 1000 person-years for ages 18–34 compared with 20.0 per 1000 person-years for ages 75–90). This divergence between relative and absolute risk is critical for informing clinical decision-making.

The investigators additionally classified participants into four exposure categories based on the co-occurrence of prediabetes and obesity: prediabetes with obesity (19.2 %), prediabetes without obesity (31.5 %), obesity without prediabetes (12.2 %), and neither condition (37.1 %). Compared with individuals free of both exposures, the combination of prediabetes and obesity conferred a 32 % increased risk of incident ASCVD, while prediabetes alone was associated with a 22 % increase and obesity alone a 10 % increase. Notably, prediabetes without obesity carried greater ASCVD risk than obesity without prediabetes, highlighting that dysglycemia is an independent driver of atherosclerotic risk rather than merely a byproduct of excess adiposity.

The size and diversity of this cohort lend the findings broad generalizability. A particularly important sensitivity analysis censored individuals who progressed to type 2 diabetes during follow-up and yielded results consistent with the primary analysis, suggesting that the observed ASCVD risk is attributable to prediabetes itself rather than merely reflecting impending progression to diabetes. This is consistent with UK Biobank data in which over two-thirds of individuals with prediabetes who developed cardiovascular or kidney disease did not progress to diabetes over 11 years of follow-up [6].

However, several limitations warrant consideration. As with any electronic health record–based study, ascertainment of prediabetes through administrative codes and single laboratory values introduces misclassification risk, and the absence of data on physical activity, dietary patterns, and family history of premature ASCVD limits the ability to fully exclude residual confounding. Perhaps most importantly, the mean follow-up of 4.1 years captures only a narrow window of risk. For younger adults in whom atherosclerosis evolves over decades, the true lifetime cardiovascular burden of prediabetes is almost certainly underestimated. Future analyses from this cohort should also incorporate heart failure as an endpoint, given the well-established links between insulin resistance, obesity, and heart failure with preserved ejection fraction and the rising burden of heart failure in the United States.

The clinical implications of this study are considerable. The data reinforce that prediabetes should be recognized not merely as a precursor to type 2 diabetes but as an independent risk factor for incident ASCVD, warranting more focused clinical attention and intervention (Fig. 1). Structured lifestyle interventions have been shown to reduce progression from prediabetes to diabetes and improve downstream cardiometabolic risk factor control. However, the effects of lifestyle interventions on ASCVD events are uncertain, with only one large trial from China demonstrating reductions in cardiovascular events over 30 years of follow-up [7]. Pharmacologic management of obesity, particularly with GLP-1 receptor agonists, can similarly prevent progression to diabetes and is associated with lower ASCVD risk, although the evidence for cardiovascular risk reduction derives predominantly from populations with either prevalent diabetes or established ASCVD. Whether these cardiovascular benefits extend upstream to individuals with prediabetes, with or without obesity, who have not yet progressed to diabetes or developed clinical ASCVD remains a critical unanswered question. The present study, by quantifying ASCVD risk associated with the prediabetes-obesity phenotype across age groups, strengthens the rationale for clinical trials examining early intervention in this population.

**Fig. 1:**
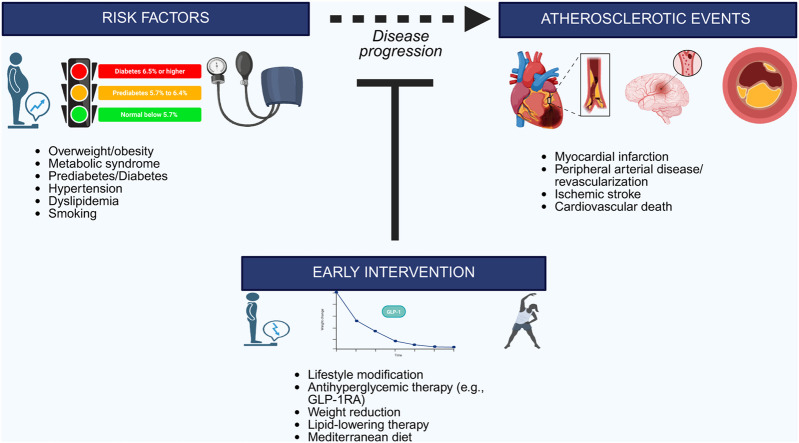
Prediabetes and Obesity as Prevention Targets: Closing the Intervention Gap. Conceptual model of atherosclerotic cardiovascular disease prevention showing the progression from risk assessment through disease development. Prediabetes and obesity represent critical intervention targets that contribute to ASCVD risk independent of progression to diabetes. Early metabolic interventions, including lifestyle modification, GLP-1 receptor agonists, weight management, and lipid-lowering therapy, may interrupt disease progression before clinical events occur.

In summary, the findings from Rana et al. add to a growing body of evidence that prediabetes, with or without concurrent obesity, is independently associated with increased ASCVD risk, particularly among younger adults. Prediabetes affects one in three U.S. adults, carries measurable cardiovascular risk, and yet prompts little clinical response. Closing this gap will require a shift from passive acknowledgment of prediabetes to active management, one that integrates metabolic risk assessment into cardiovascular prevention earlier in the disease trajectory and earlier in the lifespan.

## CRediT authorship contribution statement

**Ritika Dhruve:** Writing – review & editing, Writing – original draft, Visualization, Conceptualization. **Ambarish Pandey:** Writing – review & editing, Visualization, Conceptualization. **Neil Keshvani:** Writing – review & editing, Writing – original draft, Visualization, Conceptualization.

## Declaration of competing interest

The authors declare the following financial interests/personal relationships which may be considered as potential competing interests:

Dr. Pandey has received research support from the National Institute of Health, American Heart Association, Applied Therapeutics, Roche, Ultromics, Gilead Sciences, Bayer, and AstraZeneca; has received honoraria outside the present study as an advisor/consultant/speaker for Tricog Health Inc, Lilly, Rivus, 10.13039/100016545Roche Diagnostics, Axon Therapies, 10.13039/100006520Edward Lifesciences, Science37, 10.13039/501100004191Novo Nordisk, 10.13039/100004326Bayer, Medical AI, Astra Zeneca, Baylor Scott and White Research Institute, Boehringer Ingelheim, iRhythm Technologies, Tourmaline Bio, 10.13039/100004334Merck, Sarfez Pharmaceuticals, Ultromics, Kardigan, Tenax Pharma, Alnylam, Abbott, Kilele Health, Anumana, Acorai, Novartis, Antlia Biosciences. Dr. Keshvani has received consultant fees from 10.13039/501100016198Idorsia Pharmaceuticals, and Science37. If there are other authors, they declare that they have no known competing financial interests or personal relationships that could have appeared to influence the work reported in this paper.
